# Self-weighted dual contrastive multi-view clustering network

**DOI:** 10.1038/s41598-025-00895-6

**Published:** 2025-05-09

**Authors:** Huajuan Huang, Yanbin Mei, Xiuxi Wei, Yongquan Zhou

**Affiliations:** 1 College of Artificial Intelligence, Guangxi Minzu University, Nanning, 530006 China; 2 School of Computer Science & Technology, China University of Mining and Technology, Xuzhou, 221116 China; 3 Guangxi Key Laboratory of Hybrid Computation and IC Design Analysis, Guangxi Minzu University, Nanning, 530006 China

**Keywords:** Multi-view clustering, Deep clustering, Contrastive learning, Representation degeneration, Computational science, Computer science, Information technology, Scientific data

## Abstract

Multi-view Clustering (MVC) has gained significant attention in recent years due to its ability to explore consensus information from multiple perspectives. However, traditional MVC methods face two major challenges: (1) how to alleviate the representation degeneration caused by the process of achieving multi-view consensus information, and (2) how to learn discriminative representations with clustering-friendly structures. Most existing MVC methods overlook the importance of inter-cluster separability. To address these issues, we propose a novel Contrastive Learning-based Dual Contrast Mechanism Deep Multi-view Clustering Network. Specifically, we first introduce view-specific autoencoders to extract latent features for each individual view. Then, we obtain consensus information across views through global feature fusion, measuring the pairwise representation discrepancy by maximizing the consistency between the view-specific representations and global feature representations. Subsequently, we design an adaptive weighted mechanism that can automatically enhance the useful views in feature fusion while suppressing unreliable views, effectively mitigating the representation degeneration issue. Furthermore, within the Contrastive Learning framework, we introduce a Dynamic Cluster Diffusion (DC) module that maximizes the distance between different clusters, thus enhancing the separability of the clusters and obtaining a clustering-friendly discriminative representation. Extensive experiments on multiple datasets demonstrate that our method not only achieves state-of-the-art clustering performance but also produces clustering structures with better separability.

## Introduction

Clustering is a traditional unsupervised learning task aimed at grouping patterns (observations, data points, or feature vectors) into distinct clusters without labels^1^. Clustering plays a crucial role in various fields, such as data mining^2,3^, image processing^4,5^, bioinformatics^6,7^, and machine learning^8,9^. With the rapid development of multimedia applications, data often originates from different domains and multiple sources. For instance, images shared on social media platforms are typically accompanied by corresponding textual tags and descriptions; specific news events may be reported by multiple news agencies; and sensor signals can be decomposed in both time and frequency domains^10^. Traditional single-view clustering methods are inadequate for handling multi-view data. To better exploit the complementary and consistency among multi-view data, Multi-view Clustering (MVC) has become a hot topic in both research and practical applications.

Compared to traditional MVC methods, Deep Learning-based MVC methods (Deep-MVC) can more effectively handle non-linear and high-dimensional data. Specifically, Deep-MVC methods typically extract non-linear and high-dimensional features through view-specific autoencoders, then globally fuse the features extracted by different view autoencoders, and finally perform clustering based on the global features^11,12^. To ensure the consistency and complementary information of multi-view data, various Deep-MVC methods employ different strategies. For example, Some methods leverage subspace representations by incorporating multiple self-expressive layers between the encoder and decoder to enhance information flow. By facilitating interactions among the self-expressive layers of different views, then construct a shared common subspace, effectively capturing cross-view consistency^13^; some methods utilize self-attention mechanisms to learn complementary information across views and preserve structural consistency by maximizing the mutual information between multiple views^14^. Despite significant progress in Deep-MVC methods in recent years, But how to effectively address the conflict between view consensus information and reconstructing view-specific private information is a challenge for Deep-MVC.

To address this issue, contrastive learning-based MVC methods have been proposed. These methods enforce representation alignment across views to extract consensus information, thereby mitigating conflicts with the reconstruction of view-specific private information^15,16^. While contrastive learning-based approaches have demonstrated promising performance, their excessive emphasis on view consistency can result in representation degradation, where high-quality views are forced to conform to low-quality ones. This phenomenon ultimately limits the effectiveness of MVC, Moreover, these contrastive learning methods generally overlook the importance of inter-cluster separability, making it difficult to achieve optimal discriminative representations through clustering-friendly structures.

**Fig. 1:**
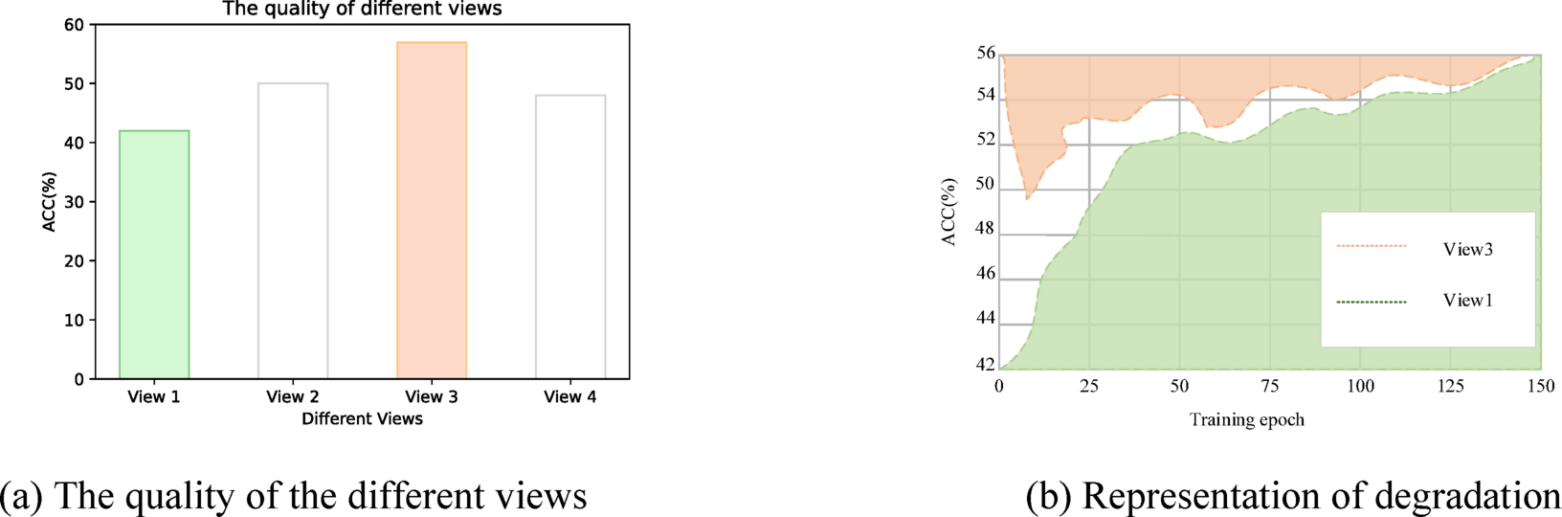
(**a**) Clustering accuracy of different views on the Cora dataset. (**b**) Clustering accuracy of view 1 and view 3 with traditional MCL method, where high quality views will be forced to be aligned with low quality views.

**Fig. 2:**
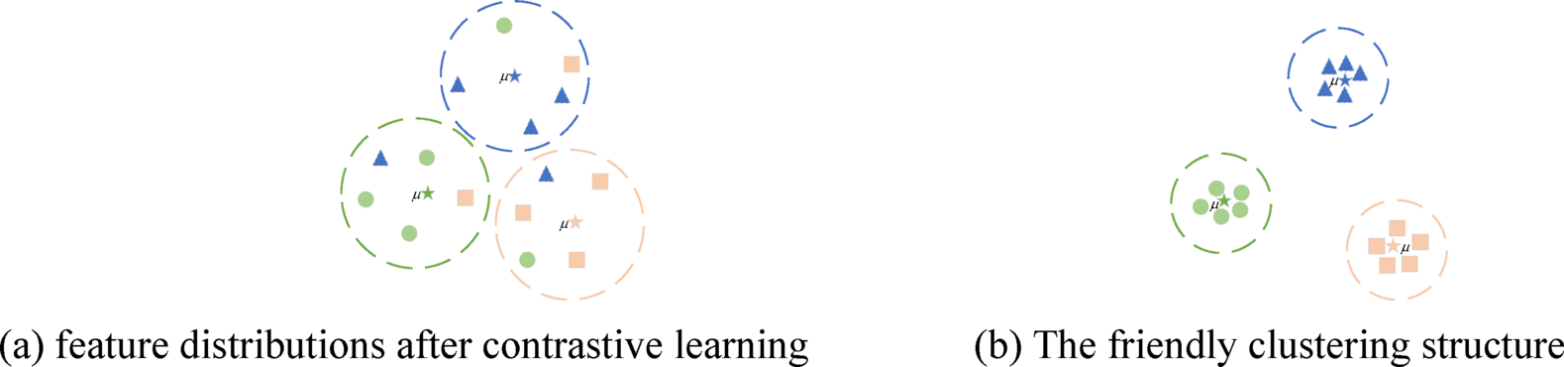
Representation of different clustering structures.

To address the aforementioned issues, we propose a novel Deep Multi-view Clustering method based on a Dual Contrastive Mechanism. Our research is driven by the following two key challenges: (1) Traditional multi-view contrastive learning (MCL) methods primarily achieve consistency by aligning cross-view latent representations. However, this approach can lead to representation degradation. Specifically, high-quality views may be forced to align with low-quality views during training (as illustrated in Fig. 1). The primary cause of this issue is the overemphasis on consistency, which overlooks the distinctive information inherent in each view. These view-specific variations often carry crucial discriminative signals, and failing to preserve them limits the model’s effectiveness. Moreover, excessive pursuit of consistency may result in the loss of valuable complementary information, thereby reducing the discriminability of the clustering process. (2) Existing MCL methods fail to learn discriminative representations with a well-structured clustering arrangement. (As shown in Fig. 2a), clusters remain partially entangled, rather than being distinctly separated. Additionally, samples within the same cluster exhibit low compactness, leading to a dispersed representation that weakens both clustering stability and discriminative capability.

Based on the above motivation, we propose an adaptive weighted view module based on contrastive learning. This module employs MLP-based view-specific encoders to extract latent representations for each view from the original data space, followed by global feature fusion to construct a unified representation. Under the assumption that representations of the same sample across different views exhibit inherent similarity, we perform multi-view contrastive learning between the view-specific latent features and the global representation to capture cross-view semantic consistency. Furthermore, by measuring the discrepancy between view-specific latent features and the global representation, we adaptively strengthen high-quality views while weakening unreliable views. This mechanism ensures that high-quality views play a dominant role in global fusion, thereby significantly mitigating the impact of unreliable views on clustering performance. To obtain discriminative representations with a cluster-friendly structure, as shown in Fig. 2b, we introduce a Dynamic cluster diffusion module(DC) from the DCMVC framework to optimize the clustering structure^12^.

Compared to existing methods, our approach offers the following contributions:

1. We propose a novel Deep Multi-view Clustering method based on a Dual Contrastive Mechanism, which effectively captures discriminative representations with a clustering-friendly structure.
2. We design an Adaptive Weighted Multi-view module, which can adaptively enhance useful views with semantic information during the feature fusion process, while suppressing the influence of unreliable views.
3. Extensive experiments are conducted on six datasets and the results demonstrate the state-of-the-art clustering performance of our proposed method.

The rest of the paper is organized as follows: Sect. 2 reviews the related work; Sect. 3 provides a detailed description of the proposed Deep Multi-view Clustering network model; Sect. 4 presents comprehensive experimental setups and result analysis; finally, Sect. 5 concludes the paper and discusses future research directions.

## Related work

In this section, we primarily review existing MVC methods and introduce Contrastive Learning, which has been widely used in many unsupervised learning tasks.

### Multi-view clustering

In this section, we classify existing MVC representation methods into three categories based on the differences in their approaches: (1) Subspace-based Multi-view Clustering: Multi-view subspace clustering methods learn a unified subspace representation from the specific subspaces of all views. In recent years, some methods have explored the potential representations of multiple views through subspace learning, effectively leveraging the complementary characteristics of multi-view data^17,18^. In addition, Su et al. proposed using anchor point learning to address the challenge of large-scale multi-view data processing^19^. Liu et al. incorporated diversity representation and block diagonal representation modules into multi-view subspaces, thereby enhancing both the discriminability and diversity of the clustering^20^. (2) Graph-based multi-view clustering: graph representation is an important data structure in multi-view data relations^21^. Many graph-based multi-view clustering algorithms are devoted to learning consensus affinity graphs across views and clustering based on them^22,23^. In order to solve the problem of different view weights, Wang et al. designed a graph fusion technique with automatically weighted graph matrices^24^. In addition, Nie et al. introduced the constrained Laplace rank (CLR), which enables the consensus affinity graph to automatically form $\:c$ connected components, thus avoiding the subsequent clustering operations^22^. (3) Multi-view clustering based on deep embedded representations: many current MVC methods are based on auto-encoder frameworks, which are deep embedded frameworks with excellent abilities to handle nonlinear features and high-dimensional data. One of the representative methods is Deep Embedded Clustering (DEC), which simultaneously learns the auto-encoder’s clustering assignment and embedding features^25^. Based on this, the improved DEC avoids the “collapse” problem of deep models by introducing a tradeoff between clustering and reconstructing objects^26^. In addition, different from the traditional self-encoder-based DEC methods, Deep Matrix Factorization (DMF) has also received much attention, which employs semi-nonnegative matrix factorization to learn the hierarchical semantics of multiple views in a hierarchical manner^27^. Subsequently, Li et al. introduced graph Laplacian regularization to the multilayer NMF model, which enabled more complex hierarchical information learning^28^.

### Contrastive learning

Contrastive learning aims to maximize the similarity between pairs of positive samples while minimizing the similarity between pairs of negative samples. Due to its excellent performance in the field of unsupervised learning, researchers have introduced contrastive Learning into the field of clustering and proposed various deep clustering methods based on contrastive learning^29^. Specifically, Li et al. construct positive-negative instance pairs via data augmentation first, and then perform contrastive learning at the instance level and cluster level respectively to jointly learn the representation and cluster allocation by simultaneously optimizing the contrastive loss at both the instance level and the cluster level^29^. Huang et al. maximize the distances between prototypical representations via contrastive learning, which improves the homogeneity of the representations, and align the positive pairs of the instances to improve intra-cluster compactness^30^. However, these methods focus on single-view data and are not applicable to multi-view data. Many multi-view clustering methods based on contrastive learning have been proposed due to its excellent performance in capturing cross-view consistency. For example, Wu et al. maximize the consensus information between global features and view-specific features through contrastive learning, which in turn effectively ensures the consistency of multi-views^11^, while Xu et al. utilize contrastive learning to achieve consistency between high-level features and semantic labels^15^. In addition, Lin et al. aligned sample representations of multi-views through contrastive learning to capture invariant information between views^31^. However, we note that most existing approaches ignore the representation degradation problem, where high-quality views are aligned to low-quality views, and also insufficiently consider the importance of maintaining cluster separability. Therefore, to address these issues, we propose a novel deep multi-view clustering method based on dual contrastive mechanism.

Compared to existing multi-view clustering methods based on contrastive learning, our approach introduces significant advancements in view-adaptive weighting and clustering structure optimization. First, to mitigate the issue of representation degradation, which is commonly observed in existing methods, we propose a contrastive learning-based view-adaptive weighting module. This module dynamically adjusts the weight of each view based on its contribution to the global representation. By effectively balancing view consistency and complementarity, our method prevents high-quality views from being forced to align with low-quality ones due to an overemphasis on consistency. In contrast, most existing methods employ fixed or heuristic weighting strategies, which fail to appropriately distinguish the importance of different views in the clustering process. Furthermore, we incorporate a Dynamic cluster diffusion module (DC) within the DCMVC framework to further refine the clustering structure. This addition enables the model to learn more compact intra-cluster representations and better-separated inter-cluster distributions, thereby improving clustering performance. In contrast, existing approaches primarily focus on instance-level contrastive learning without explicitly optimizing the clustering structure. Consequently, they often struggle to enforce well-separated clusters, resulting in suboptimal discriminative representations. By maintaining multi-view consistency while mitigating representation degradation, our method effectively overcomes the limitations of existing contrastive multi-view clustering approaches and facilitates the learning of more discriminative representations with a cluster-friendly structure.

## Proposed methods

Task Statement: Given a multi-view set $\:{\left\{{X}^{v}\right\}}_{v=1}^{M}$, which has $\:N$ samples across $\:M$ views, where $\:{X}^{v}=\left\{{X}_{1}^{v};{X}_{2}^{v}; \ldots ;{X}_{N}^{v}\right\}\in\:{R}^{N\times\:{D}_{v}}$, and $\:{D}^{v}$ represents the feature dimension of the $\:v-th$ view sample. Multi-view clustering aims to partition $\:N$ samples into $\:K$ clusters, and to improve clarity and simplicity, the primary notations used in this paper are listed in Table 1.

**Table 1:** Detailed information of notations.

Notations	Descriptions
$\:{\left\{{X}^{v}\right\}}_{v=1}^{M}$	The multi-view dataset
$\:M$	Number of views
$\:{x}_{i}^{v}$	The $\:i-th$ sample in $\:v-th$ view
$\:N$	Number of samples
$\:{D}^{v}$	Number of feature dimensions of the $\:v-th$ view data
$\:{z}_{i}^{v}$	Latent feature representation of the $\:i-th$ sample in the $\:v-th$ view
$\:\stackrel{\wedge\:}{{x}_{i}^{v}}$	The feature representation of the $\:i-th$ sample in the $\:v-th$ view reconstructed by the auto-decoder
$\:Z$	Global features which contain consistency information across views
$\:1\left[j\ne\:i\right]\in\:\left\{\text{0,1}\right\}$	Indicator function with $\:N\:$scalars
$\:{W}^{v}$	The weight of the $\:v-th$ view
$\:{\mu\:}_{k}$	The representation of the cluster center of the $\:k-th$ cluster in the global feature space
$\:{\mu\:}_{k}^{v}$	The representation of the cluster center of the $\:k-th$ cluster in the latent feature space of the $\:v-th$ view
$\:d$	Number of feature dimensions of global features

**Fig. 3:**
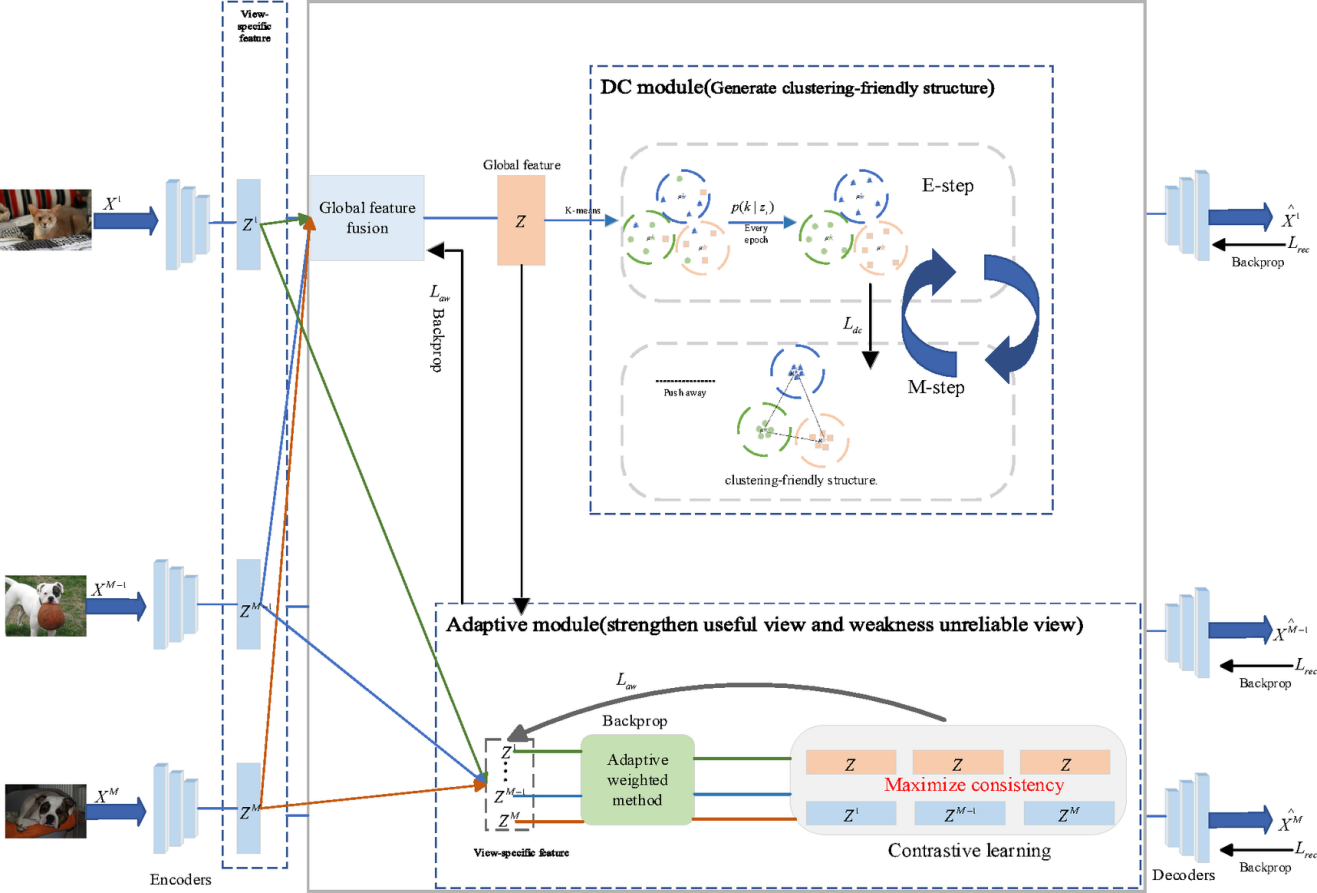
Overall network structure of the proposed method based on the expectation maximization framework.

To solve the aforementioned MVC issues, we propose a new Deep-MVC network ( details are shown in Fig. 3).

Specifically, the network mainly includes the following modules: a global feature fusion module for obtaining cross-view consistency information; an adaptive weighted module for assigning weights to different views by maximizing the consistency information of view-specific features and global features (with loss $\:{L}_{aw}$). and a dynamic cluster diffusion module to enhance inter-cluster separation by maximizing the distance between clusters (with loss $\:{L}_{dc}$).

In this section, we will focus on three modules and their optimization methods, and finally present a time complexity analysis of our methods.

### Autoencoder and global feature fusion module

In MVC, it is crucial to extract discriminative features from different features of the original multi-views. Since autoencoders perform excellent in processing nonlinear features and high-dimensional data, we utilize several view-specific autoencoders to extract the deep features of each view, while the corresponding auto-decoders are utilized to preserve other information of the original data of different views. Specifically, let $\:{f}^{v}$and $\:{g}^{v}$represent the autoencoder and auto-decoder of the $\:v-th$ view, respectively, then the latent features of the $\:v-th$ view extracted by the autoencoder can be expressed as:

1 $$\:{z}_{i}^{v}={f}^{v}({x}_{i}^{v},{\theta\:}^{v})$$

where $\:{z}_{i}^{v}\in\:{R}^{d}$ is the latent feature extracted from the $\:i-th$ sample of the $\:v-th$ view through the autoencoder, and $\:{\theta\:}^{v}$ is a parameter of the autoencoder. The decoding process of the $\:v-th$ view can be described as follows.

2 $$\:\stackrel{\wedge\:}{{x}_{i}^{v}}={g}^{v}({z}_{i}^{v},{\phi\:}^{v})={g}^{v}\left({f}^{v}\right({x}_{i}^{v},{\theta\:}^{v}),{\phi\:}^{v})$$

where $\:\stackrel{\wedge\:}{{x}_{i}^{v}}$ is the feature of the $\:i-th$ sample in the $\:v-th$ view reconstructed by the auto-decoder, and $\:{\phi\:}^{v}$ is a parameter in the auto-decoder. Similar to the traditional autoencoder, the reconstruction loss is also introduced for multi-view, which allows the autoencoder for all views to capture latent features and reduce the loss of information across views simultaneously. The reconstruction loss of the multi-view autoencoder can be described by the following equation:

3 $$\:{L}_{rec}={\sum\:}_{v=1}^{V}{\sum\:}_{i=1}^{N}{\left\| {x}_{i}^{v}-\hat x_i^v\right\|}_{2}^{2}$$

Consistency and complementary across views is highly important in the MVC process. Raw data from different views have complementary latent features extracted by view-specific autoencoders. To capture the consistency across views and obtain better clustering results, two popular approaches are concatenation and fusion^12^. Among them, concatenation is to connect features from different views into a global feature^11^, however, this approach often requires a MLP layer to downscale the global feature in subsequent work, which increases the computational complexity. Therefore, in this paper, we choose the fusion approach to obtain the global consensus information, and its expression can be represented by the following equation:

4 $$\:Z={\sum\:}_{v=1}^{V}{w}_{v}{z}_{i}^{v}$$

where$\:\:Z$ is a global feature that contains consistent information across views, and $\:{w}_{v}$ is the weights of different views, which is a parameter that can be learned. In order to solve the representation degeneration problem in view alignment, the adaptive weighted module in the follow-up work will assign weights to views based on different view qualities, as detailed in the second part of this section.

### Adaptive weighted module

In the previous section, we obtained the global feature $\:Z$ of multi-view by fusion as shown in Eq. (4), and in order to preserve the consistency between the global feature $\:Z$ and the view-specific feature $\:\{{z}^{v}{\}}_{v=1}^{M}$, we let the feature dimensions of $\:Z$ be the same as the feature dimensions of $\:\{{z}^{v}{\}}_{v=1}^{M}$. As mentioned before, indirectly obtaining consistent semantic information for multiple views by employing view feature alignment may cause a representation degradation problem, therefore, we employ multi-view contrastive learning (MCL) to obtain the consensus information of global feature $\:Z$ and view-specific feature $\:\{{z}^{v}{\}}_{v=1}^{M}$, which generates a contrastive loss can be described as:

5 $$\:{L}_{aw}={\sum\:}_{i=1}^{M}{L}_{aw}^{v}({z}^{v},Z)$$

The learnable global feature space $\:Z$ contains consistent information for each view, and the specific latent representations of the same sample in different views are similar, based on this, the representations of the same sample in global features and the specific representations in different views should be mapped closer. Therefore, we let $\:\{{z}_{j}^{v},{Z}_{i}{\}}_{j\ne\:i}^{v=1, \ldots ,M}$ as $\:M$ positive feature pairs and the rest $\:\{{z}_{j}^{v},{Z}_{i}{\}}_{i\ne\:j}^{v=1, \ldots ,M}$ as $\:M(N-1)$ negative feature pairs, meanwhile we use cosine similarity to measure the similarity between different feature pairs:.

6 $$\:sim({Z}_{i},{z}_{j}^{v})=\frac{\left\langle {Z}_{i},{z}_{j}^{v}\right\rangle}{\left\| {Z}_{i}\right\| \left\| {z}_{j}^{v} \right\| }$$

where $\left\langle \cdot \right\rangle$ denotes the dot product operation, and the specific contrastive loss is represented as follows:

7 $$\:{L}_{aw}({z}^{v},Z)=-\frac{1}{N}{\sum\:}_{i=1}^{N}{log}\frac{{e}^{sim({Z}_{i},{z}_{i}^{v})/\tau\:}}{{\sum\:}_{j=1}^{N}1\left[j\ne\:i\right]{e}^{sim({Z}_{i},{z}_{j}^{v})/\tau\:}}$$

where $\:\tau\:$ is a temperature parameter that moderates the effect of similarity and $\:1\left[j\ne\:i\right]\in\:\left\{\text{0,1}\right\}$ is an indicator function.

Several of the previous MCL works equally consider each view, ignoring the problem of view representation degeneration^12,16,32^. To address this problem, we assign different weights $\:{W}^{v}$ to different views, which adaptively adjusts the contribution of each view according to the view quality during the process of global feature fusion. Specifically, it will enhance the contrastive learning between that view’s features and the global features if a view is useful; and vice versa, it will weaken the contrastive learning between them. In this way, quality views can dominate the fusion process, which mitigates the view representation degeneration problem. Therefore, our adaptive multi-view weighted contrastive loss can be described as:

8 $${L_{aw}}=\sum\limits_{{v=1}}^{M} {{W^v}L_{{aw}}^{v}({z^v},Z)}$$

Effectively distinguishing whether view information is useful or not in MVC is tricky, and we solve this problem based on the dissimilarity between the global view feature $\:Z$ and the view-specific feature $\:\{{z}^{v}{\}}_{v=1}^{M}$. Specifically, the lower the discrepancy between the view-specific feature and the global view feature also means that they are more relevant to each other, and then the view will have a higher weight. Thus the view weights can be updated as:

9 $$\:{W}^{v}=f\left(I\right({z}^{v},Z\left)\right)$$

where $\:I(\cdot\:)$ denotes the discrepancy between specific view features and global view features, and $\:f(\cdot\:)$denotes the view weight function assigned based on the discrepancy. To estimate the correlation between different feature pairs, the maximum mean discrepancy (MMD) can effectively measure the discrepancy between two distributions P and Q. This method is based on the expectations of the two-view data, where $\:{X}_{1}=\{{x}_{1}^{i}{\}}_{i=1}^{{n}_{1}}$ and $\:{X}_{2}=\{{x}_{2}^{j}{\}}_{j=1}^{{n}_{2}}$ are generated from distributions P and Q, respectively^33^. Mathematically, MMD can be expressed as:

10 $$\:MMD({X}_{1},{X}_{2})={\left\| \frac{1}{{n}_{1}}{\sum\:}_{i=1}^{{n}_{1}}\phi\:\left({x}_{i}^{1}\right)-\frac{1}{{n}_{2}}{\sum\:}_{j=1}^{{n}_{2}}\phi\:\left({x}_{j}^{2}\right)\right\|}_{H}$$

where $\:\phi\:(\cdot\:)$ denotes the nonlinear mapping function and H denotes the Reproducing Kernel Hilbert Space(RKHS). We obtain the following equation by squaring Eq. (10)

11 $$\begin{aligned} MM{D^2} & =\left\| {\frac{1}{{{n_1}}}\mathop \sum \limits_{{i=1}}^{{{n_1}}} \varphi \left( {x_{i}^{1}} \right) - \frac{1}{{{n_2}}}\mathop \sum \limits_{{j=1}}^{{{n_2}}} \varphi \left( {x_{j}^{2}} \right)} \right\|_{H}^{2} \hfill \\ {\text{ }} & =\left\| {\frac{1}{{{n_1}}}\mathop \sum \limits_{{i=1}}^{{{n_1}}} \varphi \left( {x_{i}^{1}} \right)} \right\|_{H}^{2}+\left\| {\frac{1}{{{n_2}}}\mathop \sum \limits_{{j=1}}^{{{n_2}}} \varphi \left( {x_{j}^{2}} \right)} \right\|_{H}^{2} - 2{\left\| {\frac{1}{{{n_1}{n_2}}}\mathop \sum \limits_{{i=1}}^{{{n_1}}} \mathop \sum \limits_{{j=1}}^{{{n_2}}} \varphi \left( {x_{i}^{1}} \right)\varphi \left( {x_{j}^{2}} \right)} \right\|_H} \hfill \\ \end{aligned}$$

Here, we adopt a high-dimensional mapping $\:\phi\:\left(x\right)$ corresponding to the Gaussian kernel denoted as $\:\phi\:\left(x\right)=[{e}^{-\frac{{\left\| x-{c}_{1}\right\|}^{2}}{2{\sigma\:}^{2}}},{e}^{-\frac{{\left\| x-{c}_{2}\right\|}^{2}}{2{\sigma\:}^{2}}}, \ldots]$. specifically, when computing MMD, it is unnecessary to explicitly compute $\:\phi\:\left(x\right)$, as the Gaussian kernel inherently defines the inner product in the RKHS, thereby implicitly capturing the nonlinear feature space representation, i.e., $\:K(x,y)=\left\langle \phi\:\left(x\right),\phi\:\left(y\right) \right\rangle$ the above equation can be expanded as:

12 $$\:MM{D}^{2}=\frac{1}{{n}_{1}^{2}}{\sum\:}_{i=1}^{{n}_{1}}{\sum\:}_{j=1}^{{n}_{1}}k\left({x}_{i}^{1},{x}_{j}^{1}\right)+\frac{1}{{n}_{2}^{2}}{\sum\:}_{i=1}^{{n}_{2}}{\sum\:}_{j=1}^{{n}_{2}}k\left({x}_{i}^{2},{x}_{j}^{2}\right)-\frac{2}{{n}_{1}{n}_{2}}{\sum\:}_{i=1}^{{n}_{1}}{\sum\:}_{j=1}^{{n}_{2}}k\left({x}_{i}^{1},{x}_{j}^{2}\right)$$

from Eqs. (10,11,12) we can infer the discrepancy formula between global and view-specific pairs of views:

13 $$\begin{aligned} I\left( {{z^v},Z} \right) & =MM{D^2}\left( {{z^v},Z} \right) \hfill \\ & =\frac{1}{{{N^2}}}\mathop \sum \limits_{{i=1}}^{N} \mathop \sum \limits_{{j=1}}^{N} k\left( {z_{i}^{v},z_{j}^{v}} \right)+\frac{1}{{{N^2}}}\mathop \sum \limits_{{i=1}}^{N} \mathop \sum \limits_{{j=1}}^{N} k\left( {{Z_i},{Z_j}} \right) - \frac{2}{{{N^2}}}\mathop \sum \limits_{{i=1}}^{N} \mathop \sum \limits_{{j=1}}^{N} k\left( {z_{i}^{v},{Z_j}} \right) \hfill \\ \end{aligned}$$

Considering that the feature $\:{z}^{v}$having lower discrepancy with the global feature $\:Z$ should be assigned a higher weight value, we employ the normalization function $\:f\left(I\right({z}^{v},Z\left)\right)=Soft{max}(-I({z}^{v},Z))$ as the view weight assignment function. The view weight is updated by:

14 $$\begin{gathered} {W^v}=Soft\hbox{max} ( - I({z^v},Z)) \hfill \\ {\text{ }}=\frac{{{e^{ - I\left( {{z^v},Z} \right)}}}}{{\mathop \sum \nolimits_{{v=1}}^{M} {e^{ - I\left( {{z^v},Z} \right)}}}} \hfill \\ \end{gathered}$$

$\:{W}^{v}$ adaptively adjusts the consistency objective between $\:Z$ and $\:\{{z}^{v}{\}}_{v=1}^{M}$, where the useful views will dominant feature fusion while the unreliable views are weakened, significantly mitigating representation degeneration.

### Dynamic cluster diffusion module

In many previous MVC studies, the importance of inter-cluster separability has often been overlooked, resulting in an inability to fully exploit the clustering discriminative information through effective clustering structures^16^. To address this issue, we introduce a Contrastive Learning-based Dynamic Cluster Diffusion(DC) module^12^, which not only achieves intra-cluster cohesion across views but also facilitates the separation of clusters in the latent representation space. Specifically, the module assumes that the multi-view dataset consists of $\:k$ classes, and it obtains the feature representations of the corresponding cluster centers in both the global feature space and each view-specific feature space. Then, the following cluster-driven contrastive loss function is employed to maximize the distance between clusters:

15 $$\begin{aligned} {L_{dc}} & =\frac{1}{K}\sum\limits_{{v=1}}^{M} {\sum\limits_{{k=1}}^{K} { - \log \frac{{\exp (\frac{{s({\mu _k},\mu _{k}^{v})}}{{{\tau _c}}})}}{{\exp (\frac{{s({\mu _k},\mu _{k}^{v})}}{{{\tau _c}}})+\sum\limits_{{j=1}}^{K} {\exp (\frac{{s({\mu _k},{\mu _j})}}{{{\tau _c}}})} }}} } \\ & = - \frac{1}{K}\sum\limits_{{v=1}}^{M} {\sum\limits_{{k=1}}^{K} {\exp (\frac{{s({\mu _k},\mu _{k}^{v})}}{{{\tau _c}}})} } \\ & \quad +\frac{1}{K}\sum\limits_{{v=1}}^{M} {\sum\limits_{{k=1}}^{K} {\log (\exp (\frac{{s({\mu _k},\mu _{k}^{v})}}{{{\tau _c}}})+\sum\limits_{{j=1}}^{K} {\exp (\frac{{s({\mu _k},{\mu _j})}}{{{\tau _c}}})} )} } \\ \end{aligned}$$

Since the last term $\:{log}({exp}(\frac{s({\mu\:}_{k},{\mu\:}_{k}^{v})}{{\tau\:}_{c}})+{\sum\:}_{j=1,j\ne\:k}^{K}{exp}(\frac{s({\mu\:}_{k},{\mu\:}_{j})}{{\tau\:}_{c}}))$ contains both positive and negative sample terms, it is difficult to optimize it directly, so the calculation can be simplified by using the log-sum-exp (LSE) approximation^30^, i.e.,

16 $$\begin{gathered} \log (\exp (\frac{{s({\mu _k},\mu _{k}^{v})}}{{{\tau _c}}})+\sum\nolimits_{{j=1,j \ne k}}^{K} {\exp (\frac{{s({\mu _k},{\mu _j})}}{{{\tau _c}}})} ) \approx \log \sum\nolimits_{{j=1,j \ne k}}^{K} {\exp (\frac{{s({\mu _k},{\mu _j})}}{{{\tau _c}}})} \hfill \\ \hfill \\ \end{gathered}$$

Equation (16) is introduced into Eq. (15):

17 $$\begin{aligned} {L_{dc}} & =\frac{1}{K}\sum\limits_{{v=1}}^{M} {\sum\limits_{{k=1}}^{K} { - \log \frac{{\exp (\frac{{s({\mu _k},\mu _{k}^{v})}}{{{\tau _c}}})}}{{\exp (\frac{{s({\mu _k},\mu _{k}^{v})}}{{{\tau _c}}})+\sum\nolimits_{{j=1,j \ne k}}^{K} {\exp (\frac{{s({\mu _k},{\mu _j})}}{{{\tau _c}}})} }}} } \\ & = - \frac{1}{K}\sum\limits_{{v=1}}^{M} {\sum\limits_{{k=1}}^{K} {(\frac{{s({\mu _k},\mu _{k}^{v})}}{{{\tau _c}}})} } +\frac{1}{K}\sum\limits_{{v=1}}^{M} {\sum\limits_{{k=1}}^{K} {\log (\exp (\frac{{s({\mu _k},\mu _{k}^{v})}}{{{\tau _c}}})+\sum\nolimits_{{j=1,j \ne k}}^{K} {\exp (\frac{{s({\mu _k},{\mu _j})}}{{{\tau _c}}})} )} } \\ & \approx - \frac{1}{K}\sum\limits_{{v=1}}^{M} {\sum\limits_{{k=1}}^{K} {(\frac{{s({\mu _k},\mu _{k}^{v})}}{{{\tau _c}}})} } +\frac{1}{K}\sum\limits_{{v=1}}^{M} {\sum\limits_{{k=1}}^{K} {\log (\sum\nolimits_{{j=1,j \ne k}}^{K} {\exp (\frac{{s({\mu _k},{\mu _j})}}{{{\tau _c}}})} )} } \\ \end{aligned}$$

Here $\:{L}_{dc}$ enforces cluster cohesion (pulling pairs of positive samples closer together) in the first term and cluster separation (separating pairs of negative samples) in the second term. where $\:s({\mu\:}_{k},{\mu\:}_{j})=\frac{{\mu\:}_{k}^{T}{\mu\:}_{j}}{\left\langle {\mu\:}_{k}\right\rangle \left\langle {\mu\:}_{j}\right\rangle}$ is a measure of the similarity of the feature representations of the cluster centers, $\:{\tau\:}_{c}$ is a temperature parameter used to control the sharpness of the distributions, and $\:{\mu\:}_{k}$ and $\:{\mu\:}_{k}^{v}$ denote the feature representations of the $\:k-th$ cluster centers of the global and view-specific feature spaces, respectively, which are described by the mathematical expressions as:

18 $$\:{\mu\:}_{k}={\frac{{\sum\:}_{{Z}_{i}\in\:B}p\left(k\right|{Z}_{i}){Z}_{i}}{\left\langle{\sum\:}_{{Z}_{i}\in\:B}p\left(k\right|{Z}_{i}){Z}_{i}\right\rangle}}_{2}$$

19 $$\:{\mu\:}_{k}^{v}=\frac{{\sum\:}_{{z}_{i}^{v}\in\:\beta\:}p\left(k\right|{z}_{i}^{v}){z}_{i}^{v}}{{\left\langle{\sum\:}_{{z}_{i}^{v}\in\:\beta\:}p\left(k\right|{z}_{i}^{v}){z}_{i}^{v}\right\rangle}_{2}}$$

where $\:p\left(k\right|{Z}_{i})$ denotes the hard assignment of the $\:i-th$ sample belonging to the $\:k-th$ cluster.

During the training process, obtaining an accurate $\:p\left(k\right|{Z}_{i})$is crucial for optimizing the proposed model. Here, EM framework can be used to optimize the model, in each E-step, alternately using K-means to obtain the cluster centroid representation, and then obtain the initial hard assignment $\:p\left(k\right|{Z}_{i})$. Then in M-step, the cluster centroid representation is updated by minimizing the loss function $\:{L}_{dc}$, to optimize the model. Specifically, as the optimization progresses, the update of cluster centers no longer solely relies on the initial hard assignment but is dynamically adjusted through the loss function, introducing the property of soft assignment. This approach more accurately reflects the contribution of each sample to different clusters, allowing $\:p\left(k\right|{Z}_{i})$ to serve as a weighting factor for updating cluster centers, thereby promoting a more reasonable and robust clustering structure during training.

The aforementioned clustering-driven contrastive loss consists of two primary components, the cluster cohesion term, which facilitates interactions across views, and the cluster separation term, which promotes the separation of different clusters from each other in latent feature space, resulting in a distinct and fully separated cluster structure.

### Optimal

We train our deep clustering network in pre-training stage and fine-tuning stage. In the pre-training stage, using randomly initialized parameters for these deep autoencoders may lead to convergence of the model to a local optimum during training. Therefore, we first train these deep autoencoders to obtain better parameters $\:\{{\theta\:}^{v}{\}}_{v=1}^{M}$ and$\:\:\{{\phi\:}^{v}{\}}_{v=1}^{M}$ to speed up the convergence of the model to the optimal solution. In the fine-tuning stage, we first calculated the adaptive weight contrastive loss (Eq. (8)) to iteratively update the different view weights; then, due to the importance of obtaining an accurate $\:p\left(k\right|Z)$ for optimizing our model, we exploited the expectation-maximization (EM) framework to train our model, specifically, alternately using K-means in each E-step on the obtained global features for clustering, and then in M-step we take into account all module losses and optimize the following overall objective loss using the Adam optimizer:

20 $$\:{L}_{total}={L}_{rec}+\partial\:\cdot\:{L}_{dc}+\beta\:\cdot\:{L}_{aw}$$

where $\:\partial\:$ and $\:\beta\:$ are two hyperparameters to control the balance of the three loss components.

The training process of our proposed method is summarized in Algorithm 1, and the final clustering results are obtained by performing K-means clustering on the consensus representation Z generated by the global feature fusion module.

**Algorithm 1:**
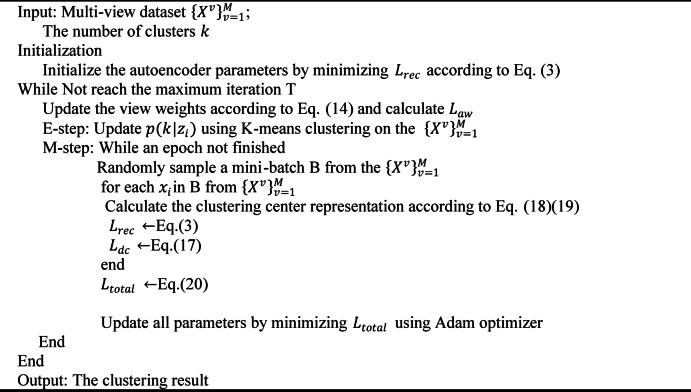
Training algorithm.

### Complexity analysis

The computational complexity of the algorithm for the model proposed in this paper includes two primary components: adaptive weighted feature fusion and dynamic cluster diffusion. On the one hand, in calculating different view weights by contrastive learning loss, the computational complexity is $\:3{N}^{2}d$. In fusing the potential features of specific views into global features based on different view weights, the computational complexity is $\:NVd$, where$\:N$denotes the number of samples, $\:V$ denotes the number of views, and d stands for the feature dimensionality of the samples. Thus, after a maximum of T iterations, the computational complexity is $\:O(3T{N}^{2}Vd+TNVd)$. On the other hand, the computational complexity of K-means clustering on E-steps is $\:NKd$, subsequently a min-batch $\:B$ is randomly sampled in M-steps assuming that its size is $\:b$. The computational complexity of the cluster center representation obtained by Eqs. (16)(17) is $\:KVb{d}^{2}$, where $\:K$ denotes the number of clusters. Furthermore one round of iteration in M-step needs to iterate Mini-batches, Mini-batches=$\:\frac{N}{b}$, so the time complexity of each round is $\:O\left(\frac{N}{b}\right(NKd+KVb{d}^{2}\left)\right)=O(\frac{{N}^{2}Kd}{b}+NKV{d}^{2})$, and the time complexity after the maximum number of $\:T$ iterations is $\:O(\frac{T{N}^{2}Kd}{b}+TNKV{d}^{2})$, so the overall time complexity is $\:O(3T{N}^{2}Vd+TNVd+TNVK{d}^{2}+\frac{{N}^{2}TKd}{b})$, Since the parameters of $\:K,V,b,d$ are far less than the number of samples $\:N$, the time complexity of the model can be be approximated as $\:O\left(T{N}^{2}d\right(3V+K\left)\right)$.

## Experiments

In this section, six datasets are selected for experiments and the results are compared with 12 representative and state-of-the-art methods to verify the effectiveness of the proposed method.

### Dataset

Our experiments are conducted on the following datasets, for details please refer to Table 2.

**Table 2:** Description of the experimental baseline dataset.

Dataset	Sample	View	Class	Dimension
RGB-D	1449	2	13	2048/300
Cora	2708	4	7	2708/1433/2708/2708
CCV	6773	3	20	5000/5000/4000
Hidigit	10,000	2	10	784/256
ALOI	10,800	4	100	77/13/64/125
Digit-product	30,000	2	10	1024/1024

1. Sentences NYU v2(RGB-D): RGB-D is a set of indoor images which includes textual descriptions^34^. For the first view, we use image features pre-extracted by the ResNet-50 network and for the second view, we use image descriptions obtained by training a doc2vec model on the Wikipedia dataset^12^.
2. Cora: Cora is a text dataset that includes seven classes^35^, and in this paper we select all four of its content, inbound, outbound, and citation features as different views.
3. Columbia Consumer Video(CCV): CCV is a video dataset that contains about 6773 samples over 20 categories^36^. We selected three hand-crafted bag-of-words representations as different views from it, which are STIP, SIFT and MFCC.
4. Hdigit: The Hdigit dataset is derived from the MNIST and USPS handwritten digit datasets and contains 10,000 samples and two different views^37^.
5. ALOI: ALOI is a subset of ALOI-1 K where the color similarity, Haralick, HSV, and RGB features are extracted separately for each image and these features are used for the four views^38^.
6. Digit-product: Digit-product is similar to Hdigit, derived from the MNIST and Fashion HandWritten datasets, and contains 30,000 samples and two views^39^.

### Compared methods and evaluation measures

We compare our proposed deep clustering method based on dual contrastive learning with the following traditional methods and some of the latest deep clustering approaches.

1. K-means: K-means is a classic clustering algorithm that partitions data by minimizing the distance between a point and the cluster centroid^40^.
2. BMVC: BMVC efficiently incorporates collaborative information from multiple views by jointly learning collaborative discrete representations and binary cluster structures^41^.
3. LMVSC: LMVSC constructs a smaller graph for the view between the raw data and the anchors via anchor learning, followed by merging these anchor graphs for subspace clustering.
4. FPMVS-CAG: FPMVS-CAG enables the two processes to collaborate with each other and improve the clustering quality by constructing a unified optimization framework for anchor selection and subspace^42^.
5. EAMC: EAMC is an end-to-end Multi-Modal Adversarial Attention Network that leverages adversarial learning to align the latent distributions and employs attentional mechanisms to quantify the importance of different modalities^43^.
6. SiMVC: Simple Multi-view Clustering (SiMVC) exhibits competitive or superior performance by prioritizing information views through a linear combination mechanism^16^.
7. DSMVC: DSMVC simultaneously extracts complementary information across views and discards meaningless noise through automatic feature selection^32^.
8. ProPos: ProPos enhances the consistency of the representation by maximizing the distance between prototypes and augments the view by sampling neighbor alignment for intra-cluster compactness^30^.
9. CoMVC: Contrastive Multi-view Clustering (CoMVC) combines a selective contrast alignment module with SiMVC to leverage alignment advantages while maintaining the priority of the information view^16^.
10. MFLVC: MFLVC effectively balances reconstruction of view-private with learning of common semantics through multi-level feature learning^15^.
11. GCFAgg: GCFAgg aligns consensus and view-specific representation information through a structure-guided contrastive learning module^44^.
12. DCMVC: DCMVC achieves intra-cluster compactness and inter-cluster separateness by introducing a dual contrastive learning module, respectively, which realizes the best clustering discriminative information through a friendly clustering structure^12^.

We selected quantitative metrics including unsupervised clustering accuracy (ACC)^45^, normalized mutual information (NMI)^46^, and purity (PUR)^47^.

### Implementation details

We adopt the Fully Connected (FC)-based layer as the main layer of the deep network, specifically, for the view-specific network, the encoder architecture is set to: input-FC500-FC500-FC2000-FC256, and the decoder is the mirror structure of each view-specific encoder. We use RELU^48^ as the activation function and Adam^49^ as the optimizer. We have a default learning rate of 0.0003, a batch size of 256, and 200 epochs of all view-specific autoencoders trained in the pre-training stage; in the training stage, the network is trained for 200 epochs on each dataset. the temperature parameter, $\:\tau\:$, is set to 1, and $\:{\tau\:}_{c}$ is set to 0.5. for the hyperparameter, $\:\partial\:$, it is chosen from the range $\:\left\{\text{0.001,0.01,0.1,1}\right\}$, and for the hyperparameter $\:\beta\:$, it is chosen in the range of $\:\left\{\text{0.4,0.6,0.8,1}\right\}$.

Our experiments are conducted on Ubuntu 22.04, and the hardware configuration includes an NVIDIA 4090 graphics processing unit (GPU), an Intel(R) Xeon(R) Silver 4310 cpu, and a 32GB of RAM.

### Experimental results

**Fig. 4:**
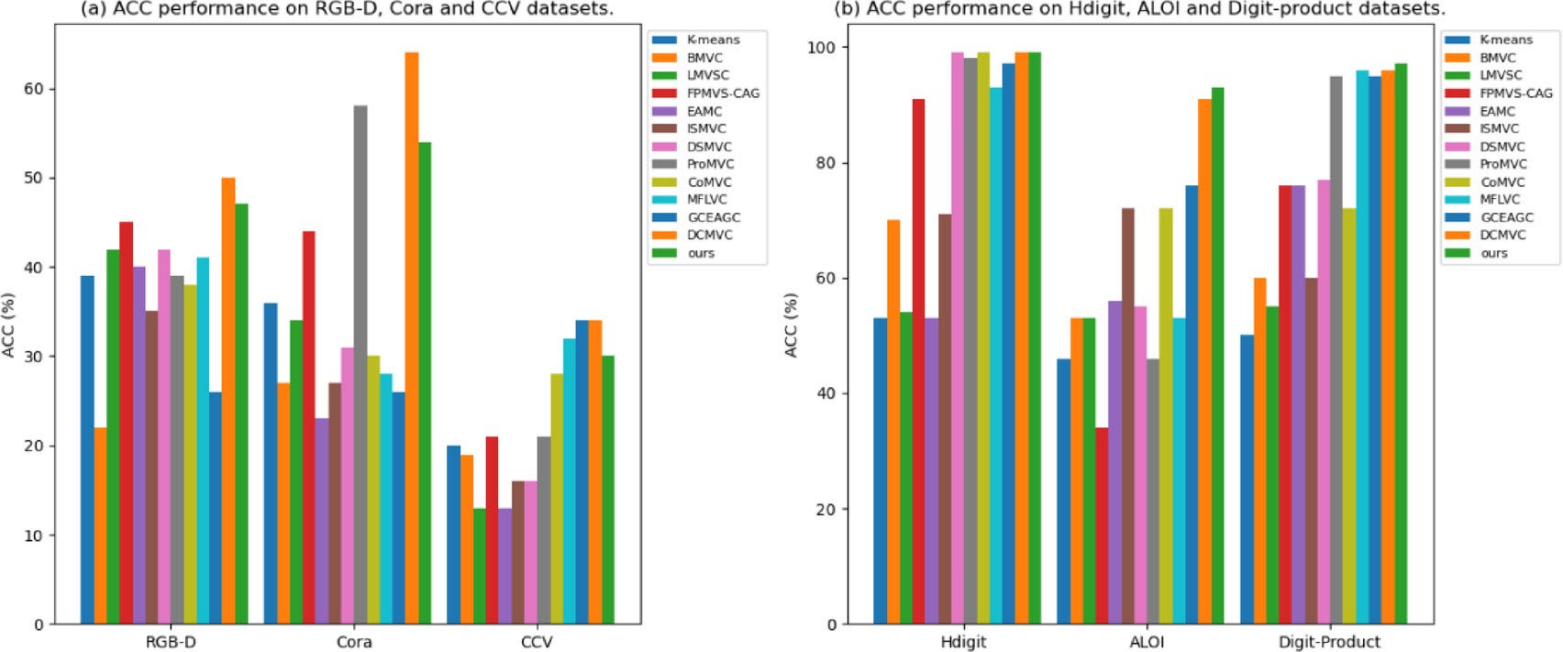
ACC’s performance on the dataset.

The results of the experiments on the six datasets are outlined in Tables 3 and 4. The ACC metrics of the comparison experiments are shown in Fig. 4. The optimal and sub-optimal results are presented in bold and underlined, respectively, and it is notable that:

1. In some small-scale datasets, our method outperforms most models. for example, on the RGB-D, Cora, and CCV datasets, it achieves significantly higher quantitative metrics in the clustering task than the traditional MVC approach. Compared to the EAMC, SIMVC, and MFLVC methods, our ACC enhances by 7.21%, 11.76%, and 5.55%, respectively, which indicates that our method performs better in capturing multi-view complementary information. However, our results are not optimal compared to some latest experimental methods for example DCMVC. The reason for this result may be that false negatives disturb the clustering process during contrastive learning.
2. In general, single-view clustering (e.g., K-means) performs inferiorly, however, we found that some multi-view clustering methods, i.e., BMVC and SIMVC, perform worse than K-means on RGB-D, Cora, and CCV datasets, which may be attributed to the fact that many MVC methods fail to efficiently extract discriminative features from multi-views, which affects the clustering performance. This observation shows that simply having multiple views is not sufficient to ensure excellent performance; the key lies in how to effectively integrate and utilize multi-view information. Our method employs a dual-contrast mechanism that is able to extract discriminative features from multiple views.
3. Our approach outperforms other models in terms of quantitative metrics obtained by clustering on the larger datasets such as Hidigit, ALOI, and Digit-product. This may be attributed to the fact that most MVC approaches equally consider each view in the clustering process, which ignores the impact of some low-quality views containing redundancy and noise on the clustering. In our method, the adaptive weighted module based on contrastive learning can adaptively strengthen the useful view and weaken the useless view, thus improving the clustering effect.

**Table 3:** Clustering results on RGB-D, Cora and CCV datasets.

Dataset	RGB-D			Cora			CCV		
Metric	ACC	NMI	PUR	ACC	NMI	PUR	ACC	NMI	PUR
K-means	0.3927	0.3690	0.5023	0.3674	0.1511	0.3818	0.1992	0.1779	0.2257
BMVC	0.2236	0.1191	0.3513	0.2718	0.0698	0.3413	0.1877	0.1651	0.2283
LMVSC	0.4160	0.4008	0.4327	0.3427	0.1336	**0.7094**	0.1313	0.0859	**0.4261**
FPMVS-CAG	0.4500	0.3489	0.5216	0.4460	0.2086	0.4460	0.2116	0.1640	0.2315
EAMC	0.3982	0.3041	0.5073	0.2315	0.0060	0.3028	0.1266	0.1011	0.1320
SIMVC	0.3527	0.3433	0.5170	0.2700	0.0817	0.3367	0.1598	0.1190	0.1864
DSMVC	0.4216	0.3658	0.5600	0.3028	0.0814	0.3579	0.1626	0.1217	0.1939
propos	0.3865	0.3702	0.5521	*0.5827*	**0.4525**	0.6199	0.2079	0.1830	0.2348
COMVC	0.3795	0.3523	0.5428	0.2999	0.0883	0.3737	0.2802	0.2731	0.3180
MFLVC	0.4148	0.2194	0.4237	0.2810	0.1310	0.3977	0.3204	0.3161	0.3594
GCFAgg	0.2622	0.2039	0.4113	0.2622	0.1087	0.3549	*0.3399*	*0.3166*	*0.3720*
DCMVC	**0.5025**	*0.4027*	**0.5963**	**0.6401**	*0.4380*	*0.6725*	**0.4296**	**0.4026**	0.3447
Ours	*0.4703*	**0.4076**	*0.5866*	0.5440	0.3810	0.6252	0.2989	0.2768	0.3461

**Table 4:** Clustering results on Hidigit, ALOI and Digit-product datasets.

Dataset	Hidigt			ALOI			Digit-product		
Metric	ACC	NMI	PUR	ACC	NMI	PUR	ACC	NMI	PUR
K-means	0.5291	0.4717	0.5473	0.4614	0.6711	0.4950	0.4992	0.4769	0.5680
BMVC	0.7040	0.5750	0.7510	0.5320	0.7200	0.5640	0.5909	0.4294	0.6321
LMVSC	0.5424	0.4736	0.5800	0.5323	0.7192	0.5323	0.5524	0.5013	0.6002
FPMVS-CAG	0.9104	0.8060	0.9104	0.3354	0.6520	0.3317	0.7560	0.6884	0.7563
EAMC	0.5268	0.7440	0.5702	0.5608	0.1799	0.0623	0.7553	0.7844	0.7733
SIMVC	0.7119	0.6727	0.7119	0.7220	0.8979	0.7354	0.6003	0.5920	0.6003
DSMVC	0.9909	0.9545	0.9909	0.5487	0.7654	0.5600	0.7656	0.6970	0.7656
propos	0.9780	0.9179	0.9780	0.4656	0.6719	0.4750	0.9523	0.9148	0.9523
COMVC	0.9872	*0.9656*	0.9872	0.7221	0.8997	0.7490	0.7183	0.6700	0.7183
MFLVC	0.9266	0.8425	0.9266	0.5290	0.8289	0.5274	0.9648	**0.9857**	*0.9688*
GCFAgg	0.9730	0.9262	0.9730	0.7631	0.8951	0.7867	0.9503	0.9387	0.9503
DCMVC	0.9920	0.9643	0.9920	*0.9129*	*0.9360*	*0.9320*	*0.9676*	*0.9529*	0.9676
Ours	**0.9926**	**0.9785**	**0.9926**	**0.9300**	**0.9415**	**0.9366**	**0.9727**	0.9531	**0.9727**

### Ablation study

In this section, we perform a detailed ablation study to gain a deeper insight into the sub-modules designed for MVC. The ablation results are shown in Table 5.

1. Effect of Adaptive weighted feature fusion: AW is an adaptive weighted module based on contrastive learning. In order to evaluate the effectiveness of adaptive weighted feature fusion introduced in our network, we compared it with a network utilizing average fusion. As shown in Table 5, our method outperforms the method with no AW in terms of clustering performance. It is evident from these results that adaptive weighted feature fusion enables the model to capture more comprehensive feature information and obtain an adaptive clustering representation compared to average fusion.
2. Effect of Dynamic Cluster Diffusion: we evaluate the effectiveness of DC module by comparing the proposed method with a degraded network with no DC module. From the results in Table 5, we can see that without the DC module, the clustering performance is degraded. For example, on the Digit-product dataset, the absence of the DC module decreases the ACC by 2.04% and the PUR by 3.11%; on the RGB-D dataset, the ACC decreases by 1.84%. These results indicate that the DC module can effectively improve the clustering performance of our network. Besides, the DC module further optimizes the clustering representation by augmenting the separability across clusters.
3. Effects of Contrastive Loss Functions: In our proposed method, it mainly involves two kinds of loss functions $\:{L}_{aw}$ and $\:{L}_{dc}$. Among them, $\:{L}_{aw}$ plays a crucial role in our method, without it, the ACC metrics of our method on the datasets RGB-D, CCV, and Digit-product will be decreased by 16.39%, 10.15%, and 21.24%, respectively, this is because the global features computed without $\:{L}_{aw}$ will be disturbed by the irrelevant information inherent in each view, which seriously affects the clustering performance. Besides, we introduce $\:{L}_{dc}$ to optimize our clustering representation, without it, the ACC metrics of our method on the datasets RGB-D, CCV, and Digit-product will decrease by 1.02%, 1.79%, and 1.11%, respectively, this is because without b, the contribution of the samples to the different clusters is not well reflected, which results in some samples incorrect clustering.

**Table 5:** Ablation studies of key components.

Dataset	RGB-D			CCV			Digit-product		
Metric	ACC	NMI	PUR	ACC	NMI	PUR	ACC	NMI	PUR
No-AW	0.4403	0.3676	0.4665	0.2719	0.236	0.2341	0.9700	0.8931	0.8523
No-DC	0.4519	0.4057	0.5856	0.2774	0.2753	0.3447	0.9523	0.9496	0.9416
No-$\:{L}_{aw}$	0.3064	0.2997	0.3023	0.1974	0.2084	0.2116	0.7603	0.6991	0.7411
No-$\:{L}_{dc}$	0.4601	0.4003	0.5847	0.2810	0.2771	0.3450	0.9616	0.9507	0.9498
Ours	0.4703	0.4076	0.5866	0.2989	0.2768	0.3461	0.9727	0.9531	0.9727

### Model analysis

#### Visualization of the clustering results

**Fig. 5:**
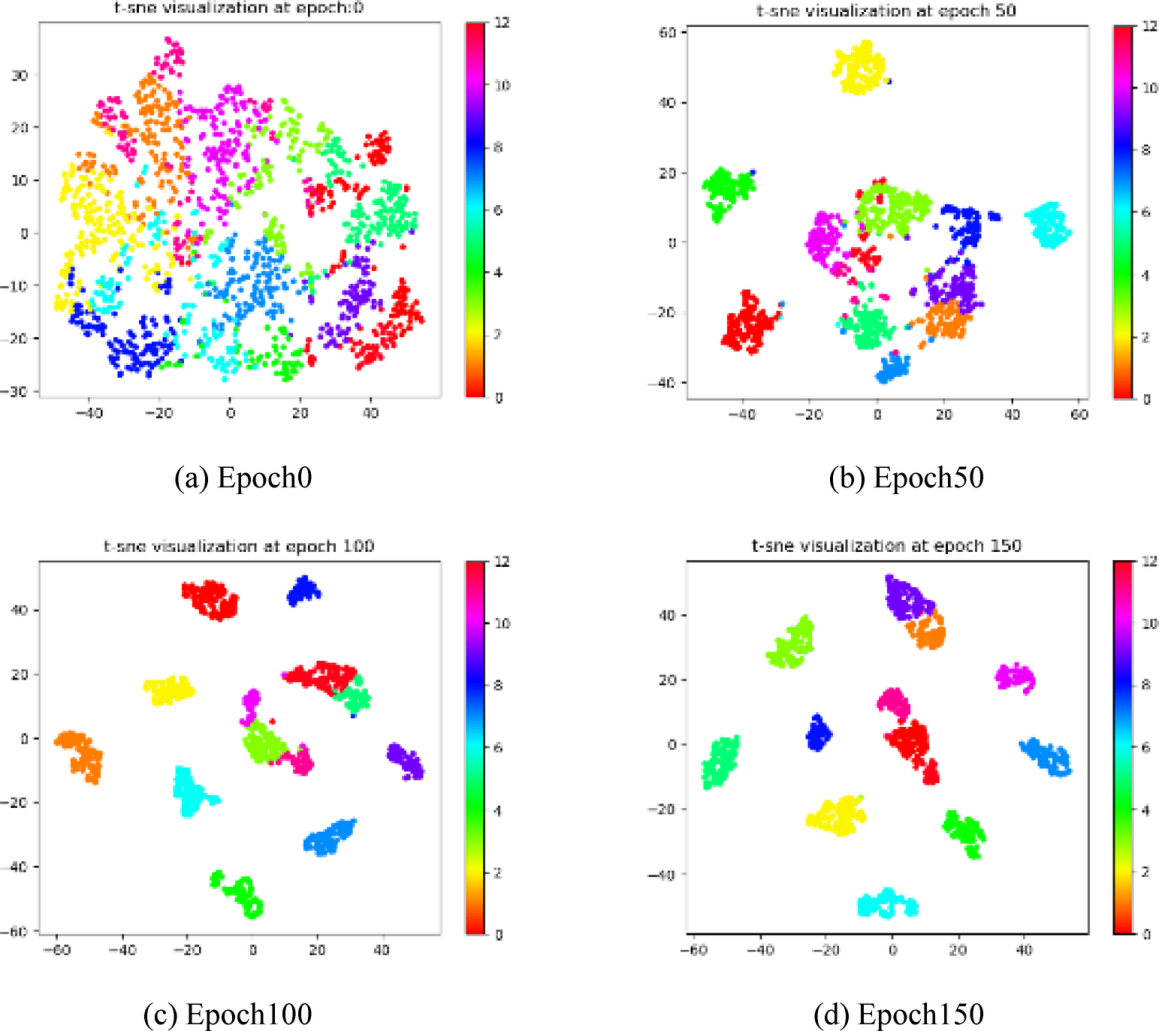
Visualization of features in RGB-D. The same color indicates features belonging to the same class.

**Fig. 6:**
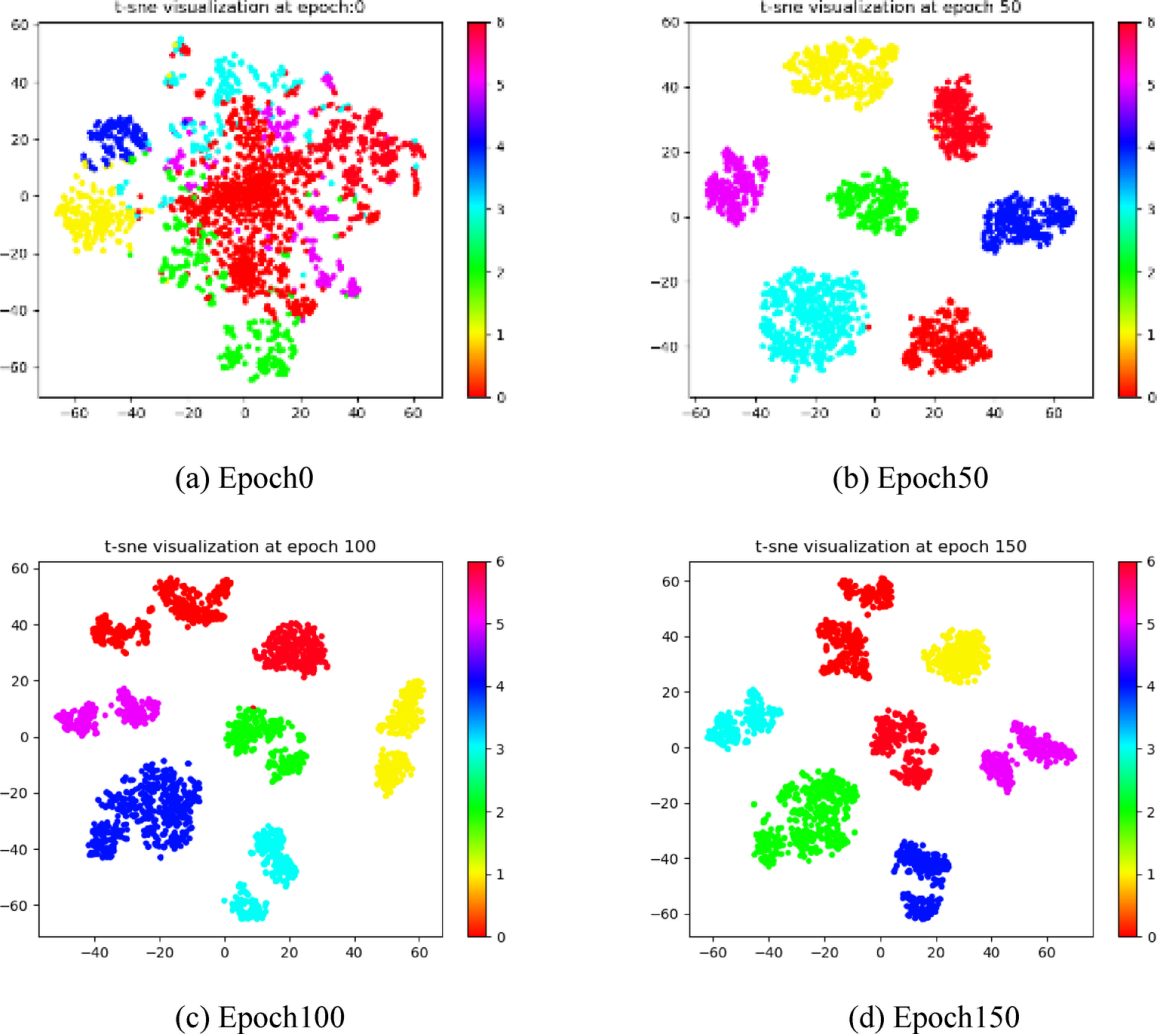
Visualization of features in Cora. The same color indicates features belonging to the same class.

In order to validate that our method can obtain discriminative representations with a clustering-friendly structure, we use the t-SNE method on the RGB-D and Cora datasets to visualize the consensus representations learned in different training steps^50^. As shown in Figs. 5 and 6, as the training epoch increases, the clustering structure of the learned consensus representation gains clarity, the distance between clusters gradually increases, and the distribution of data within clusters becomes more compact.

#### View weighting analysis

**Fig. 7:**
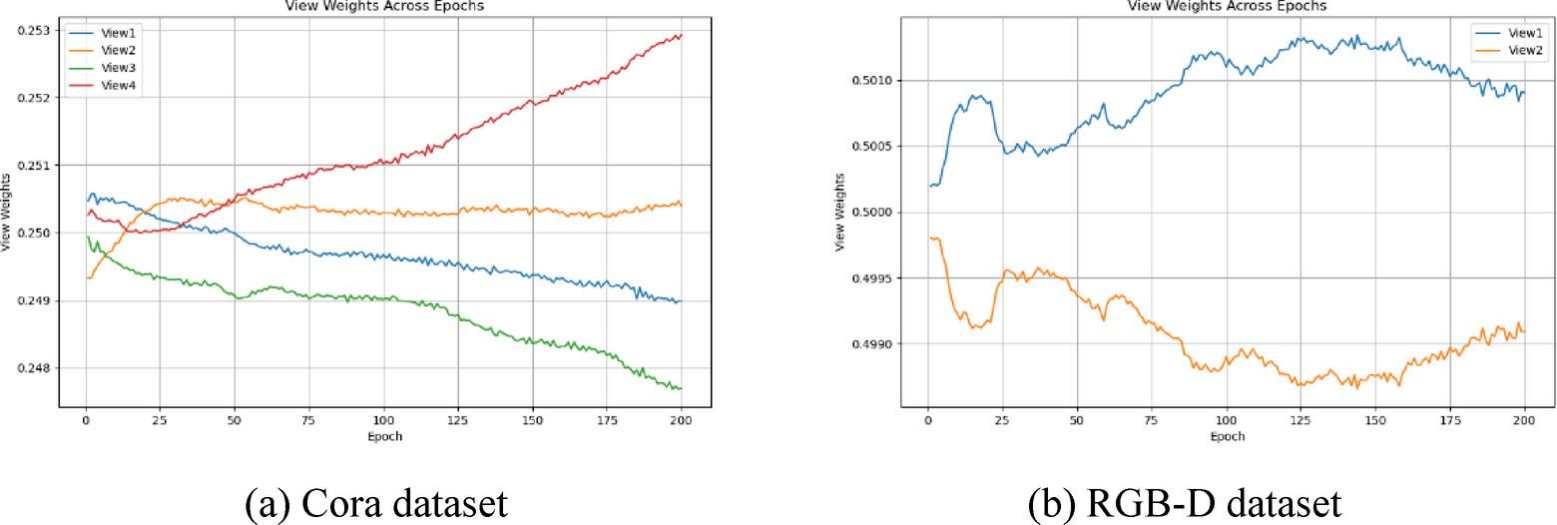
View weighting analysis on Cora, RGB-D respectively, firstly different views have the same view weights, and as the iteration increases, adaptively increase the high quality view weights and decrease the low quality view weights.

**Fig. 8:**
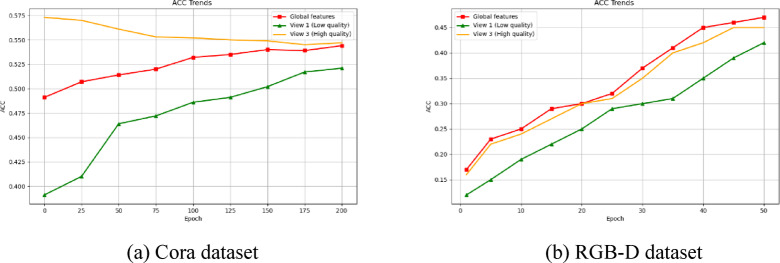
ACC of different quality views with contrastive learning iterations on Cora, RGB-D dataset, respectively. global features can learn reliable semantics from high quality views while reducing the impact of low quality views.

Adaptive weighted of views is one of the key components in our proposed method, which is designed to adaptively strengthen the weights of useful views and weaken the influence of unreliable views. This section discusses in detail how the adaptive weighted method affects the process of multi-view contrastive learning. Specifically, Fig. 7 illustrates the change trend of different view weights with the iterations on the RGB-D and Cora datasets. We observe that initially the weights of the different views are the same, but as iterations proceed, the weights of the views change based on their quality. It is notable that all views are weighted and summed to 1. High-quality views are assigned higher weights, and low-quality views are assigned lower weights. The result is that contrastive learning based on high-quality views is strengthened, while alignment errors for low-quality views are effectively mitigated. As shown in Fig. 8, the final global features can be highly consistent with the high-quality view, thus effectively extracting more useful semantic information from the reliable view. The above experimental results verify the effectiveness and advantages of our proposed adaptive weighted multi-view method.

#### Hyper-parameter analysis

**Fig. 9:**
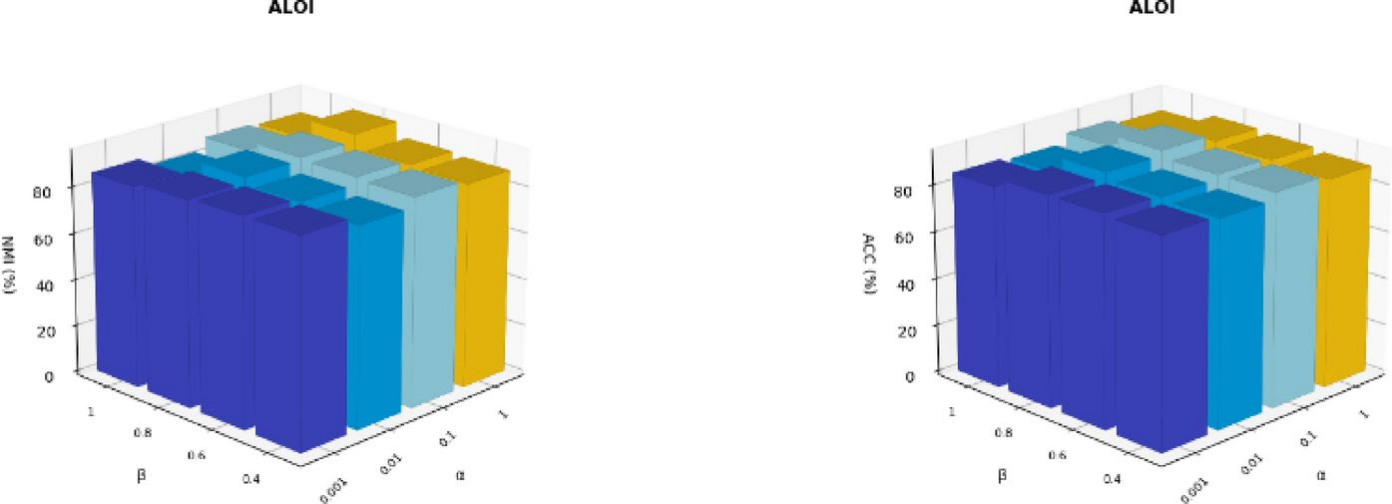
values of NMI and ACC for different combinations of $\:\partial\:$ and $\:\beta\:$ on the ALOI dataset

We conducted experiments on the ALOI dataset with various combinations of $\:\partial\:$ and $\:\beta\:$ to analyze the sensitivity of the hyperparameters. When performing sensitivity tests for a single parameter, the other parameters are fixed to their optimal values. Figure 9 present the clustering performance across different combinations of $\:\partial\:$ and $\:\beta\:$ for NMI and ACC metrics, respectively, and the experimental results show that the clustering effect of our method on the ALOI dataset remains relatively stable. In summary, our method exhibits better robustness to hyperparameter selection, maintaining consistently good performance across a variety of settings.

## Conclusion and future perspectives

In this paper, we propose a novel deep multi-view clustering network based on dual contrastive mechanism. Compared with existing MVC methods, our method can obtain a more accurate clustering representation by adaptively assigning different weights to views with different qualities, and effectively mitigates the problem of degeneration of view representations. Moreover, our method can learn discriminative representations with cluster-friendly properties, enabling better separation of different clusters in the latent space. Specifically, we introduce view-specific autoencoders for extracting the distinct features of each view. Then, we design an adaptive weighted Module based on contrastive learning to ensure that the consensus representation aligns the features of high-quality views. To further enhance the clustering effect, we introduce a dynamic cluster diffusion (DC) module. This module aims to maximize the separation between clusters by increasing the distance between different cluster representations in the consensus feature space. Experimental results show that our approach not only effectively mitigates the view representation degradation problem, but also generates better clustered discriminative representations. Experimental results demonstrate that our approach not only effectively mitigates the view representation degeneration problem, but also generates better clustering discriminative representations. Moreover, in experiments on several large-scale datasets, our method obviously better than the existing state-of-the-art methods.

In our future work, we plan to address the interference of the false-negative problem in contrastive learning on the clustering process to improve the robustness of the clustering network. We will also investigate and improve the view discrepancy formula to reduce the computational cost.

## Data Availability

All data generated or analyzed during this study are included in this published article.

## References

[1] Jain, A. K., Murty, M. N. & Flynn, P. J. Data clustering: a review. *ACM Comput. Surv. (CSUR)*. **31**(3), 264–323 (1999).

[2] Berkhin, P. A survey of clustering data mining techniques. In *Grouping Multidimensional Data: Recent Advances in Clustering* 25–71 (Springer, 2006).

[3] Neha, D. & Vidyavathi, B. M. A survey on applications of data mining using clustering techniques. *Int. J. Comput. Appl.*, **126**(2). (2015).

[4] Saxena, A. et al. A review of clustering techniques and developments. *Neurocomputing* **267**, 664–681 (2017).

[5] Oyelade, J. et al. Data clustering: Algorithms and its applications. In *2019 19th International Conference on Computational Science and Its Applications (ICCSA)*, 71–81 (IEEE, 2019).

[6] Masood, M. A. & Khan, M. N. A. Clustering techniques in bioinformatics. *IJ Mod. Educ. Comput. Sci.* **1**, 38–46 (2015).

[7] Ekanayake, J., Gunarathne, T. & Qiu, J. Cloud technologies for bioinformatics applications. *IEEE Trans. Parallel Distrib. Syst.* **22**(6), 998–1011 (2010).

[8] Zhang, Z. et al. Discriminative clustering on manifold for adaptive transductive classification. *Neural Netw.* **94**, 260–273 (2017). 28822323 10.1016/j.neunet.2017.07.013

[9] Hoya, T. Reducing the number of centers in a probabilistic neural network via applying the first neighbor means clustering algorithm. *Array* **14**, 100161 (2022).

[10] Yang, Y. & Wang, H. Multi-view clustering: A survey. *Big Data Min. Analytics*. **1**(2), 83–107 (2018).

[11] Wu, S. et al. Self-weighted contrastive fusion for deep multi-view clustering. *IEEE Trans. Multimed.* (2024).

[12] Cui, J. et al. Dual contrast-driven deep multi-view clustering. *IEEE Trans. Image Process.* (2024). 10.1109/TIP.2024.3444269 39186410

[13] Xu, K. et al. Clean and robust multi-level subspace representations learning for deep multi-view subspace clustering. *Expert Syst. Appl.* **252**, 124243 (2024).

[14] Wang, Y. et al. Consistent multiple graph embedding for multi-view clustering. *IEEE Trans. Multimedia*. **25**, 1008–1018 (2021).

[15] Xu, J. et al. Multi-level feature learning for contrastive multi-view clustering. In *Proceedings of the IEEE/CVF Conference on Computer Vision and Pattern Recognition*, 16051–16060. (2022).

[16] Trosten, D. J. et al. Reconsidering representation alignment for multi-view clustering. In *Proceedings of the IEEE/CVF Conference on Computer Vision and Pattern Recognition*, 1255–1265. (2021).

[17] Zhang, C. et al. Latent multi-view subspace clustering. In *Proceedings of the IEEE Conference on Computer Vision and Pattern Recognition*, 4279–4287. (2017).

[18] Zhang, C. et al. Generalized latent multi-view subspace clustering. *IEEE Trans. Pattern Anal. Mach. Intell.* **42**(1), 86–99 (2018). 30369436 10.1109/TPAMI.2018.2877660

[19] Su, C. et al. Anchor-based multi-view subspace clustering with graph learning. *Neurocomputing* **547**, 126320 (2023).

[20] Liu, M. et al. Multi-view subspace clustering network with block diagonal and diverse representation. *Inf. Sci.* **626**, 149–165 (2023).

[21] Zhou, L. et al. A survey and an empirical evaluation of Multi-view clustering approaches. *ACM Comput. Surveys*. **56**(7), 1–38 (2024).

[22] Nie, F., Li, J. & Li, X. Self-weighted multiview clustering with multiple graph. In *IJCAI*, 2564–2570. (2017).

[23] Mei, Y. et al. Robust graph-based multi-view clustering in latent embedding space. *Int. J. Mach. Learn. Cybernet.* 1–12. (2022).

[24] Wang, H. et al. A study of graph-based system for multi-view clustering. *Knowl. Based Syst.* **163**, 1009–1019 (2019).

[25] Xie, J., Girshick, R. & Farhadi, A. Unsupervised deep embedding for clustering analysis. *Int. Conf. Mach. Learn.* **PMLR**, 478–487 (2016).

[26] Guo, X. et al. Improved deep embedded clustering with local structure preservation. In *IJCAI*. vol. 17, 1753–1759. (2017).

[27] Zhao, H., Ding, Z. & Fu, Y. Multi-view clustering via deep matrix factorization. In *Proceedings of the AAAI Conference on Artificial Intelligence*. vol. 31, no. 1 (2017).

[28] Li, J. et al. Deep graph regularized non-negative matrix factorization for multi-view clustering. *Neurocomputing* **390**, 108–116 (2020).

[29] Li, Y. et al. Contrastive clustering. In *Proceedings of the AAAI Conference on Artificial Intelligence*. vol. 35, no. 10, 8547–8555 (2021).

[30] Huang, Z. et al. Learning representation for clustering via prototype scattering and positive sampling. *IEEE Trans. Pattern Anal. Mach. Intell.* **45**(6), 7509–7524 (2022). 10.1109/TPAMI.2022.3216454 36269906

[31] Lin, F. et al. Contrastive multi-view hyperbolic hierarchical clustering. arXiv preprint arXiv:2205.02618, (2022).

[32] Tang, H. & Liu, Y. Deep safe multi-view clustering: Reducing the risk of clustering performance degradation caused by view increase. In *Proceedings of the IEEE/CVF Conference on Computer Vision and Pattern Recognition*, 202–211. (2022).

[33] Chen, T. et al. A simple framework for contrastive learning of visual representations. In *International Conference on Machine Learning. PMLR*, 1597–1607 (2020).

[34] Kong, C. et al. What are you talking about? text-to-image coreference. In *Proceedings of the IEEE Conference on Computer Vision and Pattern Recognition*, 3558–3565 (2014).

[35] Fang, S. G. et al. Efficient multi-view clustering via unified and discrete bipartite graph learning. *IEEE Trans. Neural Networks Learn. Syst.* (2023). 10.1109/TNNLS.2023.3261460 37030820

[36] Jiang, Y. G. et al. Consumer video understanding: A benchmark database and an evaluation of human and machine performance. In *Proceedings of the 1st ACM International Conference on Multimedia Retrieval*, 1–8 (2011).

[37] Chen, M. S. et al. Representation learning in multi-view clustering: A literature review. *Data Sci. Eng.* **7**(3), 225–241 (2022).

[38] Zhang, G. Y., Huang, D. & Wang, C. D. Facilitated low-rank multi-view subspace clustering. *Knowl. Based Syst.* **260**, 110141 (2023).

[39] Xu, J. et al. Multi-VAE: Learning disentangled view-common and view-peculiar visual representations for multi-view clustering. In *Proceedings of the IEEE/CVF International Conference on Computer Vision*, 9234–9243 (2021).

[40] MacQueen, J. Some methods for classification and analysis of multivariate observations. *Proc. Fifth Berkeley Symp. Math. Stat. Probab.* **1**(14), 281–297 (1967).

[41] Zhang, Z. et al. Binary multi-view clustering. *IEEE Trans. Pattern Anal. Mach. Intell.* **41**(7), 1774–1782 (2018). 29994652 10.1109/TPAMI.2018.2847335

[42] Wang, S. et al. Fast parameter-free multi-view subspace clustering with consensus anchor guidance. *IEEE Trans. Image Process.* **31**, 556–568 (2021). 34890327 10.1109/TIP.2021.3131941

[43] Zhou, R. & Shen, Y. D. End-to-end adversarial-attention network for multi-modal clustering. In *Proceedings of the IEEE/CVF Conference on Computer Vision and Pattern Recognition*, 14619–14628 (2020).

[44] Yan, W. et al. Gcfagg: Global and cross-view feature aggregation for multi-view clustering. In *Proceedings of the IEEE/CVF Conference on Computer Vision and Pattern Recognition*, 19863–19872 (2023).

[45] Xu, W., Liu, X. & Gong, Y. Document clustering based on non-negative matrix factorization. In *Proceedings of the 26th Annual International ACM SIGIR Conference on Research and Development in Informaion Retrieval*, 267–273 (2003).

[46] Strehl, A., Ghosh, J. & Cluster, E. A knowledge reuse framework for combining multiple partitions. *J. Mach. Learn. Res.* **33**(3), 583–617 (2002).

[47] Schütze, H., Manning, C. D. & Raghavan, P. *Introduction To Information Retrieval* (Cambridge University Press, 2008).

[48] Glorot, X., Bordes, A. & Bengio, Y. Deep sparse rectifier neural networks. In *Proceedings of the Fourteenth International Conference on Artificial Intelligence and Statistics. JMLR Workshop and Conference Proceedings, 2011*, 315–323 (2011).

[49] Kingma, D. P. & Adam A method for stochastic optimization. arXiv preprint arXiv:1412.6980, (2014).

[50] Van der Maaten, L. & Hinton, G. Visualizing data using t-SNE. *J. Mach. Learn. Res.*, **9**(11). (2008).

